# Ovarian Cancer Cells Promote Glycolysis Metabolism and TLR8-Mediated Metabolic Control of Human CD4^+^ T Cells

**DOI:** 10.3389/fonc.2020.570899

**Published:** 2020-09-25

**Authors:** Wenwen Shang, Rui Xu, Ting Xu, Ming Wu, Juan Xu, Fang Wang

**Affiliations:** ^1^Department of Laboratory Medicine, The First Affiliated Hospital of Nanjing Medical University, Nanjing, China; ^2^National Key Clinical Department of Laboratory Medicine, Nanjing, China; ^3^Department of Clinical Laboratory, Taizhou People’s Hospital, Taizhou, China

**Keywords:** ovarian cancer, CD4^+^ T cells, gene expression profiles, glycolysis metabolism, toll-like receptor 8

## Abstract

An immunosuppressive microenvironment is a major obstacle for successful tumor immunotherapy. Elucidating the regulatory mechanisms of energy metabolism and functionality in CD4^+^ T cells will provide insights for the development of novel immunotherapies for ovarian cancer (OC). An Agilent microarray was used to detect differences in gene expression between peripheral CD4^+^ T cells from five OC patients and those from five healthy controls. Functional pathway analysis was performed for differentially expressed genes. Gene expression profiles revealed significant differences in expression levels of 5,175 genes in peripheral CD4^+^ T cells from five patients with OC. Functional analysis indicated that the most significantly enriched pathways were metabolic pathways. Furthermore, eight glycolysis-related genes all showed significantly increased expression in peripheral CD4^+^ T cells of OC patients. Moreover, we established a coculture system of human CD4^+^ T cells with the OC cell line SKOV3, and then treated them with toll-like receptor 8 (TLR8) ligand ssRNA 40. Coculturing with SKOV3 cells could increase the expression of the eight glycolysis-related genes, promote glucose uptake and glycolysis in CD4^+^ T cells, induce the differentiation of CD4^+^ CD25^+^ Foxp3^+^ T cells, and enhance the suppression of naïve CD4^+^ T cells. Additionally, activated TLR8 signaling could mediate the reprogramming of glycolysis metabolism and function in CD4^+^ T cells. Overall, our study indicates that the SKOV3 coculture environment could regulate the glycolysis metabolism and function of CD4^+^ T cells, and also that TLR8 mediated the metabolic control of glycolysis in CD4^+^ T cells cocultured with SKOV3 cells. This provides a new direction for immunotherapy investigations in OC.

## Introduction

Ovarian cancer (OC) is one of the most lethal malignant tumors in the reproductive system of women. Because more than 70% of patients are diagnosed at an advanced stage, most OC patients display intrahepatic metastasis or postsurgical recurrence with a 5-year survival rate lower than 40% (1). There is considerable evidence to suggest that the immune system plays a critical role in tumorigenesis (2). However, the capacity of tumor cells to establish an immunosuppressive microenvironment and evade immune surveillance is highly complex and requires further investigation. Therefore, it is important to gain a better understanding of the roles immune cells play in OC.

Accumulating data have established that the tumor microenvironment is largely orchestrated by infiltrating immune cells, including T lymphocytes, B lymphocytes, and myeloid cells. The tumor microenvironment plays a critical role in the initiation, progression, and metastasis of almost all solid tumors (3). Numerous studies have demonstrated increased populations of T cells in lung cancer, breast cancer, and hepatocellular cancer patients (4, 5). Recently, transcriptome analysis has been applied to infiltrate T cells from colon, lung, and breast cancers, which has revealed a T cell exhaustion signature and the highly suppressive nature of Treg cells (6–8).

Our previous studies have shown that the proportion and numbers of CD4^+^ T cells in peripheral blood and tumor tissues of OC patients were significantly increased, and the high number of CD4^+^ T cells was positively related to the pathological features and tumor size of OC (9, 10). However, it is unclear whether the CD4^+^ T cells from these OC patients displayed distinct transcriptional and functional features with those from healthy individuals.

Toll-like receptors (TLRs) are critical components of the human immune system that act as links between innate and adaptive immunity (11). TLRs are also very important for regulating T cell functions (12). Recent studies suggest that TLR signaling also directly regulates energy metabolism in immune cells by controlling saturated fatty acid and proinflammatory signaling, as well as by driving early glycolytic reprogramming of dendritic cells (DCs) (13). Additionally, activation of TLR1 and TLR2 signaling in mouse Treg cells increases Treg glycolysis and proliferation, and also reduces their suppressive capacity (14). Importantly, TLR8 signaling reverses the suppressive functions of human tumor-derived CD4^+^ T cells, CD8^+^ T cells, and γδ T cells (15–17). Moreover, TLR8 signaling-mediated reprogramming of glucose metabolism and function in human Treg cells can enhance anti-tumor immunity *in vivo* in a melanoma model (18, 19). However, whether TLR8 signaling can regulate the glycolysis metabolism of human CD4^+^ T cells from the OC microenvironment is still unknown.

In this study, high-throughput screening was used to investigate changes in gene expression between peripheral CD4^+^ T cells from OC patients and those from healthy controls. We identified the underlying molecular changes in CD4^+^ T cells and the potential signaling pathway mechanisms in OC patients. We also elucidated the impact of TLR8 signaling in mediating metabolism and function of CD4^+^ T cells cocultured in an OC microenvironment.

## Materials and Methods

### Patients and Specimens

This research was authorized by the Ethical Committee of the First Affiliated Hospital of Nanjing Medical University (Nanjing, China) (Ethics review No: 2017-SRFA-064), and written informed consents were obtained from all patients. Fresh peripheral blood samples were obtained from OC patients and benign ovarian tumor (BOT) patients treated at the First Affiliated Hospital of Nanjing Medical University from November 2017 to December 2018. Twenty patients with newly diagnosed OC (mean age of 60.3 ± 8.9 years) and 15 new patients with BOT (mean age of 49.5 ± 9.7 years) were enrolled in this study. None of these patients had received prior treatment before collecting specimens, and there were no other known medical conditions, especially diabetes. Peripheral blood samples from 15 age-matched healthy donors (mean age of 53.3 ± 8.3 years) who underwent a physical examination with no family history of autoimmune diseases or tumors or diabetes were enrolled as healthy controls (HC). All pathological types were confirmed by histopathology, and among the 20 OC patients, 17 patients were high-grade serous adenocarcinoma of ovary and 3 patients were clear cell carcinoma of ovary. The 15 benign ovarian tumor patients comprised 5 cases of mucinous cystadenoma, 6 cases of mature cystic teratoma, and 4 cases of ovarian thyroid cyst.

### Blood Samples Collection and Peripheral CD4^+^ T Cell Isolation

Fresh peripheral blood was collected from 20 patients with OC, 15 patients with BOT, and 15 age-matched HC. Peripheral blood mononuclear cells (PBMCs) were isolated by Ficoll-Paque PLUS density gradient centrifugation (GE Healthcare Bio-Sciences, Sweden). CD4^+^ T cells were then separated by CD4 positive isolation kit (Miltenyi Biotec, Germany). Isolated CD4^+^ T cells purity was higher than 95% as determined by flow cytometry.

### Microarray Data Production and Analysis

Total RNA containing small RNA was extracted from CD4^+^ T cells from 5 patients with OC and 5 HC by using Trizol reagent (Invitrogen). The RNA quality, integrity, and purity were measured using a bioanalyzer (2100 Bioanalyzer; Agilent Technologies, Santa Clara, CA, United States). Gel electrophoresis demonstrated that each processed RNA had a 28s/18s > 2.0 and 260/280 nm absorbance >1.8, indicating that the samples were suitable for microarray analysis. Total RNA (1 μg) was reverse transcribed into cDNA and then into labeled cRNA. The appropriate amount of cRNA was hybridized to the Agilent whole human genome 8 × 60 K microarray chip. All microarray experiments were performed at the microarray facility of CapitalBio Corporation (Beijing, China), and gene expression workflow was performed according to the manufacturer’s recommendations (Agilent Technologies). Data analysis was conducted using GeneSpring GX software (Agilent Technologies). The *t*-test was used to identify genes that were differentially expressed between CD4^+^ T cells from OC patients and HC. Criteria for selecting differentially expressed genes (DEGs) were fold change (FC) >2.0 and *P*-values < 0.05. Functional analysis was compiled using gene ontology (GO) and the Kyoto Encyclopedia of Genes and Genomes (KEGG) analyses.

### RNA Extraction and Real-Time PCR

Total RNA, extracted from CD4^+^ T cells using RNeasy Micro Kit (Qiagen, United States), was applied to reverse-transcript into cDNA with a PrimeScript RT Reagent Kit (TaKaRa Bio, Japan). Expression levels of glycolysis-related genes were analysis with ABI 7500 real-time PCR (Applied Biosystems) by applying SYBR Green. The sequences of primers were listed in Table 1.

**TABLE 1:** The sequence of primers for 8 glycolysis-related genes.

Genes	Forward primer (5′→3′)	Reverse primer (5′→3′)
mTOR	CGGACTATGACCACTTGACTC	CCAAACCGTCTCCAATGAAAGA
HIF1α	CCATTAGAAAGCAGTTCCGC	TGGGTAGGAGATGGAGATGC
GLUT1	TTGGCTCCGGTATCGTCAAC	GCCAGGACCCACTTCAAAGA
GPI	AGGCTGCTGCCACATAAGGT	AGCGTCGTGAGAGGTCACTTG
ENO1	TCATCAATGGCGGTTCTCA	TTCCCAATAGCAGTCTTCAGC
PKM2	GCCGCCTGGACATTGACTC	CCATGAGAGAAATTCAGCCGAG
LDHα	CCAGCGTAACGTGAACATCTT	CCCATTAGGTAACGGAATCG
PDK1	CTGTGATACGGATCAGAAACCG	TCCACCAAACAATAAAGAGTGCT
β-actin	GAGCTACGAGCTGCCTGACG	GTAGTTTCGTGGATGCCACAG

### Coculture of SKOV3 and CD4^+^ T Cells

SKOV3 cells were cultured in six-well plates in 2 mL McCoy’s 5A medium (Invitrogen) with 10% FBS for 24 h. For synchronization, CD4^+^ T cells were isolated from PBMCs using the CD4-positive isolation kit (Metenyi), achieving a purity that was basically higher than 95%. SKOV3 and CD4^+^ T cells (1:5) were then cocultured using the inner wells (0.4 μm pore size; Corning Costar, Corning, NY, United States) to separate the cell types. Specifically, SKOV3 cells (2 × 10^5^/well) were incubated in the lower well in 2 mL RPMI 1640 medium with 10% AB serum (Gibco), and CD4^+^ T cells (1 × 10^6^) were grown in the inner wells with or without SKOV3 cells (for controls) in 1 mL of the same medium. After 5 days of incubation, CD4^+^ T cells were washed and collected.

### Simulation TLR8 Signaling With Ligands

CD4^+^ T cells were dispensed into a 96-well plate (Corning Costar, Cambridge, MA, United States) at 3 × 10^5^ cells per well. The cells were incubated with TLR8 ligands ssRNA40 (3 μg/ml), TLR8 control ligands ssRNA41 (3 μg/ml) (InvivoGen, San Diego, CA, United States), or 1640 Medium. CD4^+^ T cells were collected after 24 h stimulation.

### Glucose Uptake and Glycolysis Assays

CD4^+^ T cells were cocultured with SKOV3 for 5 days or treated with ssRNA40 or ssRNA41 for 24 h. Glucose uptake was measured by Glucose Uptake Colorimetric Assay Kit (Biovision). L-lactate concentrations in cell culture supernatants from CD4^+^ T cells were determined using the Glycolysis Cell-Based Assay Kit (Cayman). The CD4^+^ T cells and culture supernatant was collected and glucose uptake and L-lactate concentrations determined according to the manufacturer’s protocols.

### Flow Cytometry Analysis

The fluorochrome-conjugated monoclonal antibodies used in this study were FITC-conjugated anti-CD4, APC-conjugated anti-CD25, and PE-conjugated anti-Foxp3 (all from BD Biosciences, San Jose, CA, United States). Flow cytometry analysis for CD4, CD25, and Foxp3 staining was performed according to the manufacturer’s instructions. Fluorescence data were collected on a BD FACSCalibur (BD Biosciences, San Jose, CA, United States) and analyzed with FlowJo software (Tree Star, Ashland, OR, United States).

### Proliferative Activity Assay

CD4^+^ T cells (3 × 10^5^/well) were seeded in 96-well plates, [^3^H] thymidine was added at a final concentration of 1 μCi/well, and cultured for an additional 16 h. The proliferative response of CD4^+^ T cells was tested by the incorporation of [^3^H] thymidine, which was measured with a liquid scintillation counter.

### Proliferation Assay of Naïve CD4^+^ T Cells

Naïve CD4^+^ T cells were purified from healthy donors by using Naïve CD4^+^ T cell isolation Kit II, human (Miltenyi). CD4^+^ T cells that were cultured alone or with SKOV3 and treated with TLR8 ligand ssRNA 40 or not were added at ratios of 1:0, 1:1, and 0:1 to Naïve CD4^+^ T cells in U bottomed 96-well plates. PBMCs (5 × 10^4^/well) irradiated by 40 Gy were added to the culture as antigen presenting cells (APCs) containing anti-CD3 and anti-CD28 antibody (1 μg/ml, eBioscience, San Diego, CA, United States). After 56 h of culture, [^3^H] thymidine was added at a final concentration of 1 μCi/well and cultured for an additional 16 h. The proliferative activity of Naïve CD4^+^ T cells was detected by the incorporation of [^3^H] thymidine, which was measured with a liquid scintillation counter.

### Statistical Analysis

Data were analyzed with SPSS (Statistical Package for the Social Science) 20.0 software (IBM Corp, Armonk, NY, United States) and are expressed as the mean ± standard error (SEM). Comparison with groups was calculated using Student’s *t*-test. *P-*values less than 0.05 were considered statistically significant.

## Results

### Transcriptome Analysis Suggests Metabolic Pathways Are Closely Associated With Peripheral CD4^+^ T Cells of OC Patients

**FIGURE 1:**
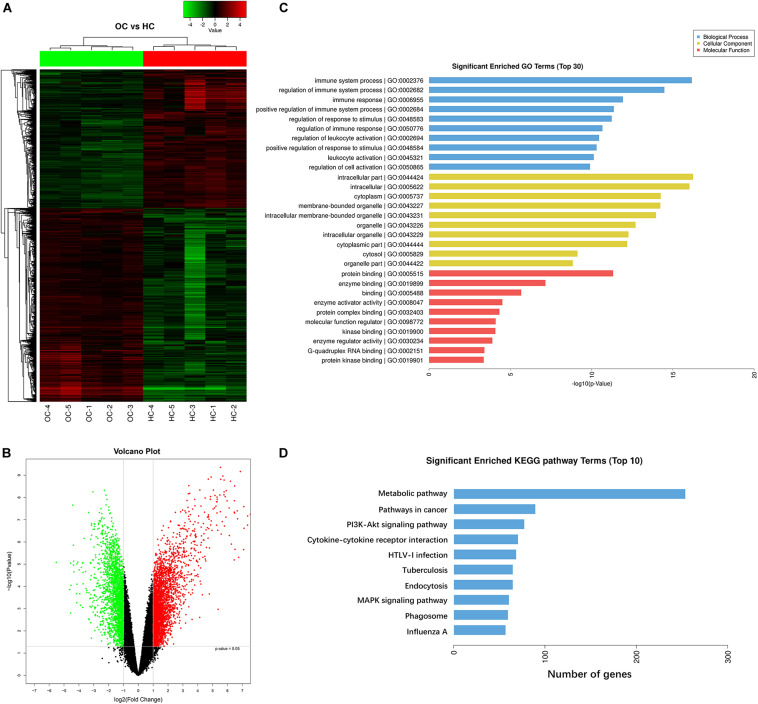
Transcriptome analysis suggests metabolic pathways are closely associated with peripheral CD4^+^ T cells of OC patients. **(A,B)** Gene expression profiles of CD4^+^ T cells derived from peripheral blood of ovarian cancer patients and healthy controls. Hierarchical clustering analysis of 5,175 mRNAs that are differentially expressed in peripheral blood CD4^+^ T cells from five ovarian cancer patients (OC) and peripheral blood CD4^+^ T cells from five healthy controls (HC), and 3,014 were up-regulated and 2,161 were down-regulated. Each column represents an individual sample and each row represents a specific gene. The threshold of significance was defined by *P* < 0.05 with Student *t*-test and the false discovery rate, log2 fold change (>2 or <–2), were considered as up-regulated and down-regulated genes, respectively. The expression values are shown in shades of red and green. Green means gene expressed at lower levels; red means gene expressed at higher levels. **(C,D)** Pathway analysis showing the significant pathways of the differentially expressed genes (*P* < 0.05 with Student *t*-test), metabolic pathways were the most significant. Gene ontology (GO) analysis indicated that the most significant enriched GO Terms on biological process among the top 10 enriched pathways were the immune system process. Kyoto Encyclopedia of Genes and Genomes (KEGG) analysis identified 296 enriched pathways, and metabolic pathways were the most significantly represented among the top 10 enriched pathways.

In this study, CD4^+^ T cells were extracted from the peripheral blood of five OC patients and five healthy volunteers. An Agilent microarray was used to detect the differential gene expression profiles of peripheral CD4^+^ T cells from five OC patients and those from five healthy controls. The total transcriptome of each sample was analyzed. As schematically illustrated in Figures 1A,B, differential expression profiles of the CD4^+^ T cells were obtained, and we detected significant differences in the expression signatures of the two groups. These showed that 5,175 mRNAs were differentially expressed in CD4^+^ T cells, of which 3,014 were up-regulated and 2,161 were down-regulated. As shown in Figures 1C,D, we used the differentially expressed mRNAs to further analyze the pathways they were significantly associated with using the Gene Ontology (GO) and Kyoto Encyclopedia of Genes and Genomes (KEGG) pathways. GO analysis indicated that the most significant enriched GO Terms on biological process among the top 10 enriched pathways were the immune system process. Further, we identified 296 enriched pathways that could be mapped using the KEGG signaling database. Metabolic pathways were the most significantly represented among the top 10 enriched pathways as shown in Figure 1D.

### Enhanced Expression of Glycolysis-Related Genes in Peripheral CD4^+^ T Cells of OC Patients

**FIGURE 2:**
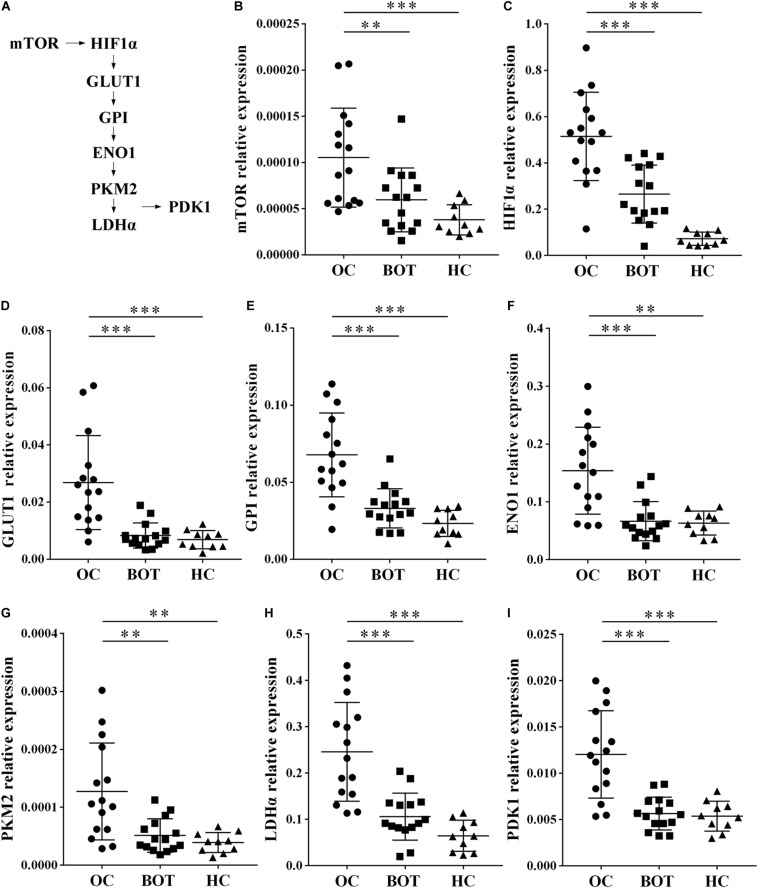
Enhanced expression of glycolysis-related genes in peripheral CD4^+^ T cells of OC patients. **(A)** Diagram of the glycolytic pathway, including the eight glycolysis genes (mTOR, HIF1α, GLUT1, GPI, ENO1, PKM2, LDHα, and PDK1) selected for further validation. **(B–I)** Gene expression levels of mTOR, HIF1α, GLUT1, GPI, ENO1, PKM2, LDHα, PDK1 in peripheral CD4^+^ T cells of OC patients (*n* = 15), BOT patients (*n* = 15), and healthy controls (*n* = 10). The expression of these eight glycolysis-related genes were all significantly higher in OC patients than in either BOT patients or healthy controls. Data are shown as mean ± SEM of three independent experiments; ^∗∗^*P* < 0.01, ^∗∗∗^*P* < 0.001.

Gene ontology and KEGG analysis indicated that differentially expressed genes in CD4^+^ T cells between OC patients and healthy individuals are most particularly associated with metabolic pathways. We therefore shifted our research focus onto these metabolic pathways. In the pathway shown in Figure 2A and Supplementary Figure 1, mammalian target of rapamycin (mTOR) activity enhances the expression of hypoxia-inducible factor 1α (HIF-1α), which stimulates glucose transport and glycolysis by upregulating key downstream enzymes, including glucose transport 1 (GLUT1), glucose-6-phosphate isomerase (GPI), enolase 1 (ENO1), pyruvate kinase muscle 2 (PKM2), lactate dehydrogenase α (LDHα), and pyruvate dehydrogenase kinase1 (PDK1). These enzymes encompass the glycolytic pathway. To validate our microarray results, we examined the expression of eight glycolysis-related genes (mTOR, HIF1α, GLUT1, GPI, ENO1, PKM2, LDHα, and PDK1) in peripheral CD4^+^ T cells from OC patients (*n* = 15), BOT patients (*n* = 15), and healthy controls (*n* = 10) by RT-PCR. As shown in Figures 2B–I, eight glycolysis-related genes including mTOR, HIF-1α, GLUT1, GPI, ENO1, PKM2, LDHα, and PDK1 were all expressed at significantly higher levels in OC patients than in either BOT patients or healthy controls (all *P* < 0.05). These results validated our microarray findings that the expression levels of glycolysis-related genes are increased in the peripheral CD4^+^ T cells of OC patients.

### OC Cells Promote the Glycolysis Metabolism of Peripheral CD4^+^ T Cells

**FIGURE 3:**
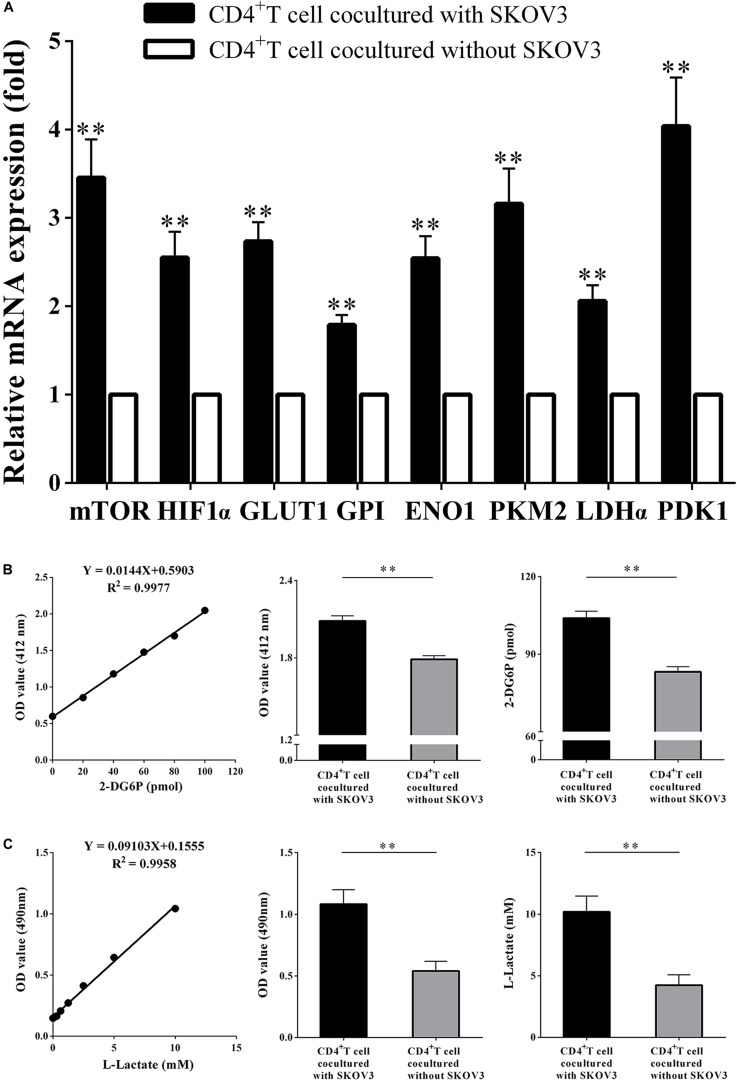
Ovarian cancer cells promote the glycolysis metabolism of peripheral CD4^+^ T cells. **(A)** The real-time PCR analysis of the mTOR, HIF1α, GLUT1, GPI, ENO1, PKM2, LDHα, and PDK1 mRNA expression in the CD4^+^ T cells cocultured with or without SKOV3. Compared with CD4^+^ T cells not cocultured with SKOV3 cells, the expression levels of eight glycolysis-related genes in CD4^+^ T cells cocultured with SKOV3 cells were all significantly higher. **(B)** The CD4^+^ T cells in the coculture system with SKOV3 expressed higher levels of glucose uptake than that in the CD4^+^ T cells cocultured without SKOV3. Glucose uptake was detected by colorimetry. **(C)** The levels of glycolysis were significantly increased in the CD4^+^ T cells cocultured with SKOV3 compared with that in the CD4^+^ T cells cocultured without SKOV3. Glycolysis was detected by colorimetry; ^∗∗^*P* < 0.01.

We next investigated the effect of OC cells on the glycolysis metabolism and function of CD4^+^ T cells. CD4^+^ T cells isolated from fresh peripheral blood of healthy volunteers were cocultured with the OC cell line SKOV3 for 5 days. As shown in Figure 3, we found that compared with CD4^+^ T cells not cocultured with SKOV3 cells, the expression levels of eight glycolysis-related genes in CD4^+^ T cells cocultured with SKOV3 cells were all significantly higher (all *P* < 0.01): mTOR (3.458 ± 0.4329 vs. 1.000), HIF-1α (2.551 ± 0.2931 vs. 1.000), GLUT1 (2.736 ± 0.2159 vs. 1.000), GPI (1.788 ± 0.1161 vs. 1.000), ENO1 (2.542 ± 0.2533 vs. 1.000), PKM2 (3.162 ± 0.4009 vs. 1.000), LDHα (2.060 ± 0.1777 vs. 1.000), and PDK1 (4.038 ± 0.5526 vs. 1.000). We further detected the glucose metabolism level of CD4^+^ T cells from healthy volunteers and found that the levels of glucose uptake (2.086 ± 0.03955 vs. 1.789 ± 0.02800; 103.9 ± 2.747 vs. 83.22 ± 1.945, *P* < 0.05) and glycolysis (1.083 ± 0.1166 vs. 0.5410 ± 0.07793; 10.19 ± 1.281 vs. 4.235 ± 0.8560, *P* < 0.05) were also significantly increased in CD4^+^ T cells cocultured with SKOV3 cells compared with those cocultured without SKOV3 cells. Thus, our data revealed that the tumor microenvironment of OC could promote the glycolysis metabolism of human CD4^+^ T cells.

### OC Cells Upregulate the Percentages of Treg Cells and Enhance the Suppressive Functions of CD4^+^ T Cells

**FIGURE 4:**
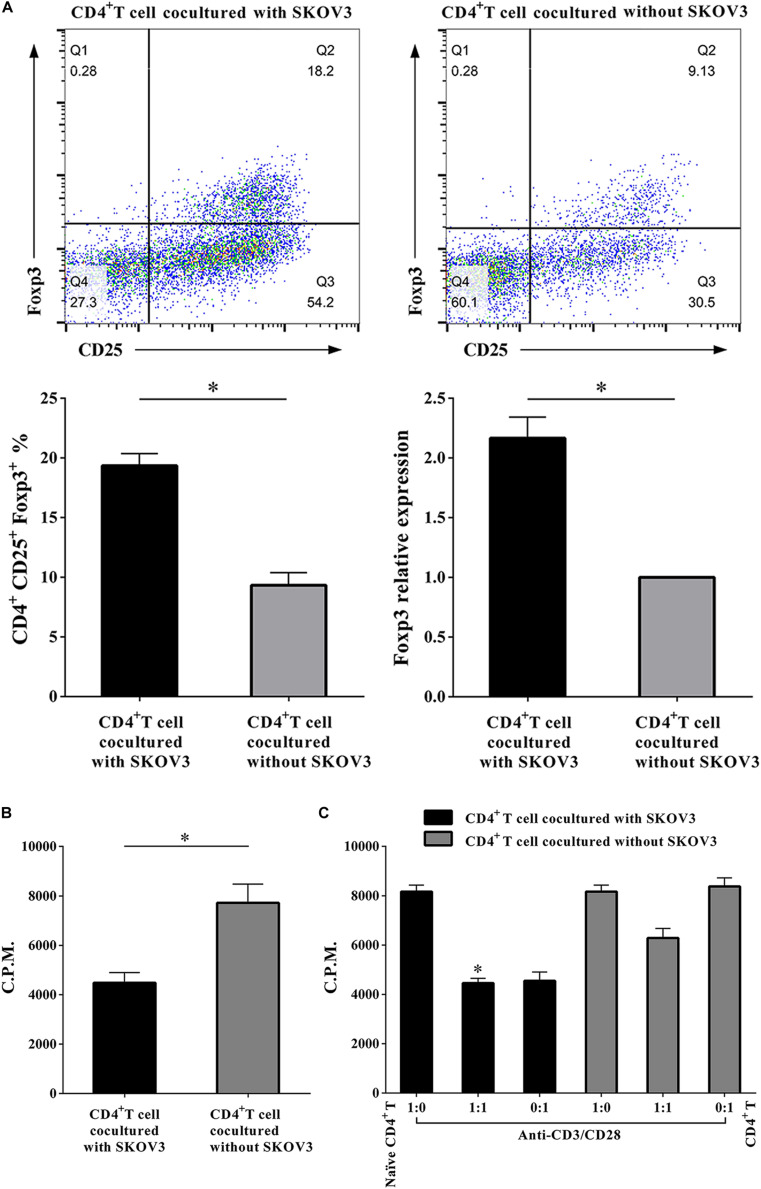
Ovarian cancer cells upregulate the percentages of Treg cells and enhance the suppressive functions of CD4^+^ T cells. **(A)** The CD4^+^ T cells cocultured with SKOV3 showed an increased expression of CD4^+^ Treg marker Foxp3 compared with that in the CD4^+^ T cells cocultured without SKOV3. **(B)** The proliferation levels of CD4^+^ T cells cocultured with SKOV3 were lower than that cocultured without SKOV3, the proliferative level was determined by [^3^H] thymidine uptake, C.P.M. is an abbreviation for counts per minute. **(C)** The suppressive ability of the CD4^+^ T cells cocultured with SKOV3 was enhanced compared with the CD4^+^ T cells cocultured without SKOV3, the proliferative response was determined by [^3^H] thymidine uptake with anti-CD3/CD28 stimulation. The data are presented as the mean ± SEM of three independent experiments; ^∗^*P* < 0.05.

More recent studies have suggested that metabolic regulation of T cell differentiation, Foxp3 expression, T cell lineage stability, and homeostasis all involve glycolysis. As shown in Figure 4, to determine if metabolic processes are critical for CD4^+^ T cell functions, we further investigated the proliferation rates of CD4^+^ T cells, the proportion of Treg cells in the CD4^+^ T cell population, and the suppressive capacity on naïve CD4^+^ T cells. We determined that the proportion of CD4^+^ CD25^+^ Foxp3^+^ T cells was markedly increased (19.34 ± 1.035 vs. 9.340 ± 1.047, *P* < 0.05), while the proliferation rates of CD4^+^ T cells were significantly decreased (4485 ± 412.6 vs. 7714 ± 764.6, *P* < 0.05) in CD4^+^ T cells cocultured with SKOV3 cells relative to those not cocultured with SKOV3 cells. Additionally, the suppressive effect of CD4^+^ T cells cocultured with SKOV3 cells on the proliferation of naïve CD4^+^ T cells (4455 ± 199.1 vs. 6263 ± 383.8, *P* < 0.05) was also enhanced compared with the CD4^+^ T cells not cocultured with SKOV3 cells. These data revealed that the glycolysis metabolism reprogramming of CD4^+^ T cells can induce the differentiation of CD4^+^ T cells into CD4^+^ CD25^+^ Foxp3^+^ Treg cells, as well as inhibit the proliferation of naïve CD4^+^ T cells.

### TLR8 Signaling Inhibits the Glycolysis Metabolism of Peripheral CD4^+^ T Cells

**FIGURE 5:**
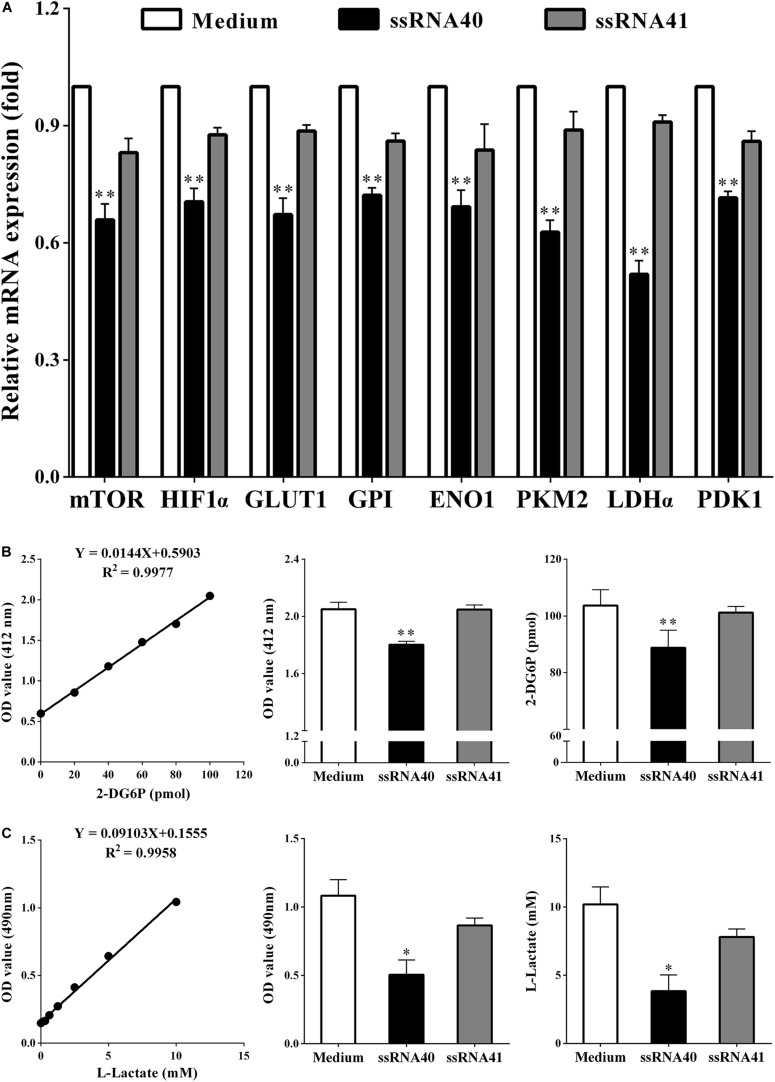
TLR8 signaling inhibits the glycolysis metabolism of peripheral CD4^+^ T cells. **(A)** The mRNA expression levels of the mTOR, HIF1α, GLUT1, GPI, ENO1, PKM2, LDHα, and PDK1 in the CD4^+^ T cells were analyzed by real-time PCR. CD4^+^ T cells were collected and treated with TLR8 ligand ssRNA40, TLR8 control ligand ssRNA41, or control Medium for 24 h after cocultured with SKOV3. Compared with CD4^+^ T cells treated with the TLR8 control ligand ssRNA41 or control medium, the expression levels of eight glycolysis-related genes (mTOR, HIF-1α, GLUT1, GPI, ENO1, PKM2, LDHα, and PDK1) in coculturing CD4^+^ T cells treated with TLR8 ligand ssRNA40 were all significantly decreased. **(B)** The levels of glucose uptake in CD4^+^ T cells treated with TLR8 ligand ssRNA40 were lower than that in the CD4^+^ T cells treated with TLR8 control ligand ssRNA41 or control Medium for 24 h. Glucose uptake was detected by colorimetry. **(C)** The levels of glycolysis were significantly decreased in the CD4^+^ T cells treated with TLR8 ligand ssRNA40 compared with that in the CD4^+^ T cells treated with TLR8 control ligand ssRNA41 or control medium for 24 h. Glycolysis was detected by colorimetry; ^∗^*P* < 0.05 and ^∗∗^*P* < 0.01.

A new study showed that TLR8 signaling could mediate the glucose metabolism of tumor-derived Treg cell lines, which resulted in Treg function reversal (18). We therefore explored whether TLR8 signaling can also affect glycolysis metabolism of CD4^+^ T cell cocultured with SKOV3 cells on the molecular level after stimulation. As shown in Figure 5, coculturing CD4^+^ T cells were treated with TLR8 ligand ssRNA40, TLR8 control ligand ssRNA41, or control medium for 24 h. We found that, compared with CD4^+^ T cells treated with the TLR8 control ligand ssRNA41 or control medium, the expression levels of eight glycolysis-related genes (mTOR, HIF-1α, GLUT1, GPI, ENO1, PKM2, LDHα, and PDK1) in coculturing CD4^+^ T cells treated with TLR8 ligand ssRNA40 were all significantly decreased (*P* < 0.05). The levels of glucose uptake (2.050 ± 0.04789 vs. 1.802 ± 0.02338; 103.7 ± 5.596 vs. 88.80 ± 6.199, *P* < 0.05) and glycolysis (1.083 ± 0.1166 vs. 0.5050 ± 0.1079; 10.19 ± 1.281 vs. 3.839 ± 1.185, *P* < 0.05) were also decreased. These data suggested that activation of TLR8 signaling can markedly affect glycolysis metabolism in human CD4^+^ T cells cocultured with OC cells.

### TLR8 Activation Downregulates the Percentage of Treg Cells and Weakens Suppressive Functions

**FIGURE 6:**
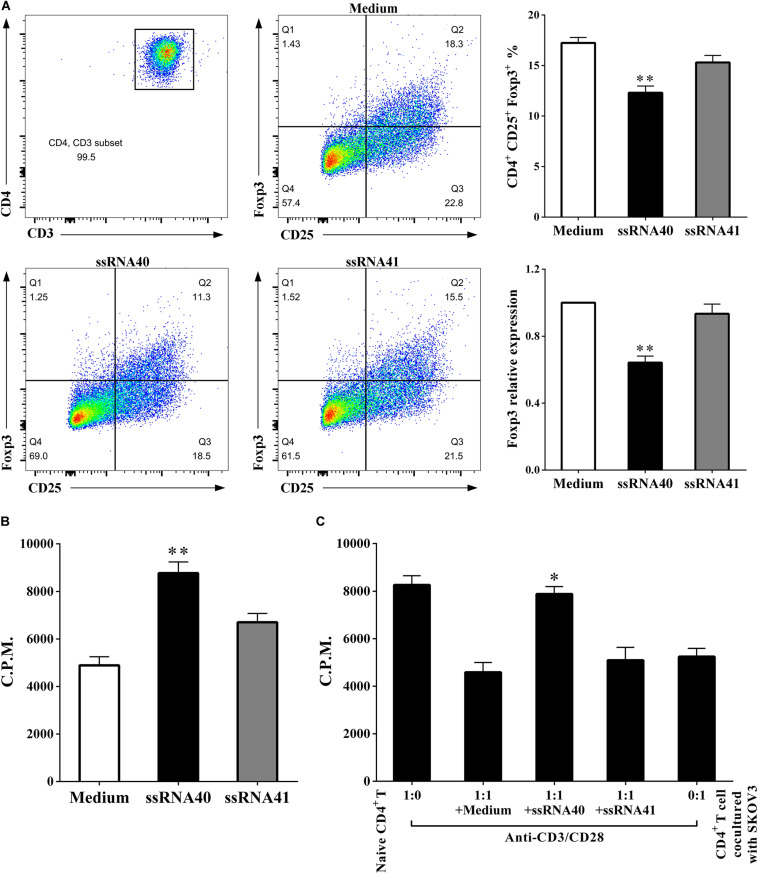
TLR8 activation downregulates the percentage of Treg cells and weakens suppressive functions. **(A)** The prevalence of Foxp3^+^ cells in the CD4^+^ T cells treated with TLR8 ligand ssRNA40 was decreased compared with that in the CD4^+^ T cells treated with TLR8 control ligand ssRNA41 or control medium. **(B)** The proliferation levels were increased in the CD4^+^ T cells treated with TLR8 ligand ssRNA40 compared with that in the CD4^+^ T cells treated with TLR8 control ligand ssRNA41 or control medium, the proliferative level was determined by [^3^H] thymidine uptake, C.P.M. is an abbreviation for counts per minute. **(C)** The suppressive effect of the cocultured CD4^+^ T cells was weakened with the treated of TLR8 ligand ssRNA40, while the treated of TLR8 control ligand ssRNA41 or control medium did not affect the suppressive ability of the cocultured CD4^+^ T cells. The proliferative response was determined by [^3^H] thymidine uptake with anti-CD3/CD28 stimulation. The data are presented as the mean ± SEM of three independent experiments. ^∗^*P* < 0.05 and ^∗∗^*P* < 0.01.

It has been demonstrated that human TLR8 signaling can reverse the suppressive functions of naturally occurring Treg cells and tumor-derived CD4^+^, CD8^+^, and γδ Treg cells, which resulted in enhanced anti-tumor immunity (18). As shown in Figure 6, we conducted analyses of coculturing CD4^+^ T cells after TLR8 ligand ssRNA40 treatment. We found that, compared with the CD4^+^ T cells treated with TLR8 control ligand ssRNA41 or control medium, the proportion of CD4^+^ CD25^+^ Foxp3^+^ T cells was significantly decreased (17.24 ± 0.5558 vs. 12.32 ± 0.6616, *P* < 0.05) in the CD4^+^ T cells treated with TLR8 ligand ssRNA40. The proliferation rates of CD4^+^ T cells were significantly increased (4894 ± 364.4 vs. 8765 ± 477.4, *P* < 0.05), as well as the suppressive effect on the proliferation of naïve CD4^+^ T cells (4588 ± 408.9 vs. 7883 ± 307.9, *P* < 0.05), in the CD4^+^ T cells treated with TLR8 ligand ssRNA40. These studies identified that TLR8 signaling can improve the proliferation of CD4^+^ T cells and decrease the proportion of CD4^+^ CD25^+^ Foxp3^+^ Treg cells, which promotes the proliferation of naïve CD4^+^ T cells.

## Discussion

Based on the evolving understanding of molecular immunology, the tumor immune microenvironment has been extensively studied (20). Accumulating evidence shows that increased numbers of T lymphocytes in cancer patients are associated with the occurrence and development of various cancers, including breast cancer, colorectal cancer, lung cancer, and others (5). Our previous work has demonstrated that CD4^+^ T cells are distributed at higher numbers in the peripheral blood and tumor tissues of OC patients and are positively related to clinicopathological parameters (9). There remain numerous challenges and gaps in knowledge remaining with the complex tumor immune microenvironment, including an understanding of the role of tumor-infiltrating T cells.

Transcriptome analysis of T cells allows for detailed study of these cells in the highly complex tumor microenvironment (21). It has been reported that distinct transcriptomic profiles of Tregs derived from human colorectal carcinoma, breast cancer, and liver cancer tissues may be important for T cell functions in homeostasis and immune suppression. These mRNA expression patterns can be used as a source for elucidating mechanisms of systemic immune dysfunction (6–8). In the current study, the mRNA expression profiles of peripheral CD4^+^ T cells isolated from OC patients were compared with those of healthy volunteers. Over 3,000 mRNAs were up-regulated and more than 2,000 mRNAs were down-regulated (Figures 1A,B). GO functional enrichment and KEGG pathway analyses were also performed, and we discovered that the most significant enriched biological process was immune system process and the most significantly enriched signaling pathways were metabolic pathways (Figures 1C,D). Recent reports suggest that distinct metabolic programs support the differentiation of T cells into separate functional subsets (22). It is largely accepted that Th1, Th2, and Th17 effector cells utilize higher rates of glycolysis, while Tregs exhibit higher levels of glycolysis and lipid metabolism than other T cell subsets (23, 24). Recent studies report that the tumor microenvironment affects T cell fate and immune responses by altering cellular metabolic processes (25, 26). Increasing glycolysis in CD4^+^ T cells results in enhanced effector functions, such as interferon γ (IFN-γ) production, while decreased glycolysis has been shown to inhibit both IFN-γ and interleukin 17 (IL-17) production (27, 28). Therefore, we became interested in the metabolic pathways after our data showed that they were the most significantly enriched pathways in CD4^+^ T cells of OC patients (Figure 1D). It has been reported that the mTOR-HIF-1α signaling pathway plays a critical role in regulating glucose uptake and glycolysis, and therefore subsequently directs cell growth and proliferation (29, 30). The activity of mTOR enhances HIF-1α expression and thereby stimulates glucose transport and glycolysis via upregulation of key downstream enzymes that encompass the glycolytic pathway, including GLUT1, GPI, ENO1, PKM2, LDHα, and PDK1 (31). Thus, we focused our analysis on eight glycolysis metabolism genes: mTOR, HIF-1α, GLUT1, GPI, ENO1, PKM2, LDHα, and PDK1. We determined that the expression levels of these eight glycolysis-related genes were all significantly higher in the peripheral CD4^+^ T cells of OC patients than in those from BOT patients or healthy controls (Figure 2).

These results lead us to further explore whether OC cells could affect the metabolism of CD4^+^ T cells and the role they play in the tumor milieu of OC. In our study, we established an *in vitro* coculture system to explore the effect of the OC microenvironment on CD4^+^ T cells. We observed overexpression of the abovementioned eight glycolysis-related genes and an upregulation of both glucose uptake and glycolysis in CD4^+^ T cells cocultured with the OC cell line SKOV3 (Figure 3). Increased aerobic glycolysis is a cardinal feature of most tumor cells that fuel their own growth and proliferation (32). Interestingly, activated T lymphocytes undergo a metabolic switch similar to cancer cells, and they upregulate aerobic glycolysis to permit proliferation and differentiation into specialized effector T cells (33, 34). Given the similarities in the metabolic profiles and nutrient requirements of cancer cells and T cells, it is possible that the abnormally high metabolic and nutrient consumption rates of tumor cells result in competition with neighboring T cells. This would lead to T cell metabolic prostration underlying their functional exhaustion. We hypothesize that OC cells may influence their own differentiation and proliferation by regulating glycolysis and metabolism of CD4^+^ T cells. Our results suggest that the OC microenvironment can inhibit the proliferation of CD4^+^ T cells, promote an increased population of CD4^+^ CD25^+^ Foxp3^+^ Treg cells, and also inhibit the proliferation of naïve CD4^+^ T cells (Figure 4). Treg cells exhibit higher levels of glycolysis and lipid metabolism than other T cell subsets, and increased glucose metabolism resulting from GLUT1 expression leads to an accumulation of Treg cells (35). Conversely, expression of Foxp3 can promote the respiratory function of Treg cells. These cells have a stronger glycolysis ability than Teff cells (36, 37). Our results suggest that OC cells can promote the glycolysis metabolism of peripheral CD4^+^ T cells. The glycolysis metabolism reprogramming of CD4^+^ T cells can induce the differentiation of CD4^+^ T cells into CD4^+^ CD25^+^ Foxp3^+^ Treg cells, as well as inhibit the proliferation of naïve CD4^+^ T cells. Thus, Tregs with elevated rates of glycolytic activity may better assist tumors elude immunosurveillance. *In vitro* cocultured human CD4^+^ T cells were utilized in this study, which may have metabolic differences compared with primary CD4^+^ T cells in the tumor microenvironment of fresh human OC tissue samples. Thus, more studies with human CD4^+^ T cells directly purified from fresh tumor tissues from OC patients should be performed in the future to explore the generality and applicability of this novel mechanism and therapeutic strategy.

Modulating the metabolic program of T cells is a promising strategy to improve the efficacy of immunotherapies (38, 39). Several molecular signaling pathways and molecules have been identified that are critical and required for T cell metabolic programming and development, including AKT-mTOR signaling, TLR signaling, and autophagy, as well as transcription factors HIF-1α, cMyc, and Foxp3 (37, 40). TLRs are critical components of the human immune system that act as links between innate and adaptive immunity. They are vital for regulating T cell functions (41). Recent studies suggest that TLR signaling also directly regulates energy metabolism in immune cells. Gerriets et al. reported that activation of TLR1 and TLR2 signaling in mouse Treg cells could increase Treg glycolysis and proliferation and reduce their suppressive capacity, while it could not reverse the suppressive function of human Treg cells (42, 43). Additionally, TLR8 signaling can reverse the suppressive functions of human tumor-derived CD4^+^ T cells, CD8^+^ T cells, and γδ T cells, which resulted in enhanced anti-tumor immunity. TLR8 signaling can also inhibit glucose uptake and glycolysis in human naturally occurring Treg cells, leading to reversal of human Treg suppression and selective effects on Treg function (14, 18). Furthermore, our data indicated that the metabolic profiles of CD4^+^ T cells derived from OC patients are different from those of healthy subjects (Figure 1). However, whether TLR8 signaling can mediate the metabolism of CD4^+^ T cells cocultured in the OC microenvironment has not yet been fully explored. In this study, we showed that TLR8 signaling significantly inhibits the expression of key glycolysis-related enzymes, including mTOR, HIF-1α, GLUT1, GPI, ENO1, PKM2, LDHα, and PDK1 (Figure 5A). Our glucose metabolomic analyses further suggested that activation of TLR8 signaling can markedly suppress glucose uptake and glycolysis in human CD4^+^ T cells cocultured with OC cells (Figures 5B,C). These *in vitro* studies identified that TLR8 signaling can improve the proliferation of CD4^+^ T cells and decrease the proportion of CD4^+^ CD25^+^ Foxp3^+^ Treg cells, which promotes the proliferation of naïve CD4^+^ T cells (Figure 6). These results support the feasibility of targeted T cell metabolism and function, and TLR8 signaling may be a novel immunotherapeutic strategy to treat human cancers. However, the potential mechanistic crosstalk between TLR8 and mTOR signaling in CD4^+^ T cells needs further study. Additionally, our studies have not been validated in an *in vivo* tumor suppressive microenvironment. Thus, our future studies will focus on whether TLR8-mediated reversal of CD4^+^ T cell functions *in vivo* is caused by the regulation of glycolysis metabolism. We are currently establishing a suitable human TLR8 transgenic mouse model, which is essential for future studies to explore the translational potential of this immunotherapeutic strategy.

Taken together, our study identified the mRNA expression profiles of peripheral CD4^+^ T cells in human OC patients, and metabolic pathways were most enriched. The CD4^+^ T cells/SKOV3 cells coculture microenvironment model helped elucidate the regulation of glycolysis metabolism and functions of CD4^+^ T cells in OC. TLR8 signaling mediated the metabolic control of glycolysis in CD4^+^ T cells cocultured with SKOV3 cells, which provided a new direction for immunotherapy investigations in OC.

## Data Availability Statement

The original contributions presented in the study are included in the article/Supplementary Material, further inquiries can be directed to the corresponding author/s.

## Ethics Statement

The studies involving human participants were reviewed and approved by the Ethical Committee of the First Affiliated Hospital of Nanjing Medical University (Nanjing, China) (Ethics Review No: 2017-SRFA-064). The patients/participants provided their written informed consent to participate in this study.

## Author Contributions

WS, RX, and TX contributed equally to this work. WS performed the experiments, analyzed data, and wrote the manuscript. RX and TX wrote the manuscript and provided essential materials. MW and JX wrote the manuscript. FW designed and supervised the study. All authors read and approved the final manuscript.

## Conflict of Interest

The authors declare that the research was conducted in the absence of any commercial or financial relationships that could be construed as a potential conflict of interest.

## Supplementary Material

SUPPLEMENTARY FIGURE 1 Diagram of the HIF-1 signaling pathway. Diagram of the HIF-1 signaling pathway by Kyoto Encyclopedia of Genes and Genomes analysis. Blue means gene expressed at lower levels. Red means gene expressed at higher levels. Green means gene expressed with no significant difference.
