# Supplementary figures and images for: Ovarian Cancer Cells Promote Glycolysis Metabolism and TLR8-Mediated Metabolic Control of Human CD4+ T Cells
Source: Front Oncol. 2020 Sep 25;10:570899. doi: 10.3389/fonc.2020.570899 (PMC7545320; doi:10.3389/fonc.2020.570899)

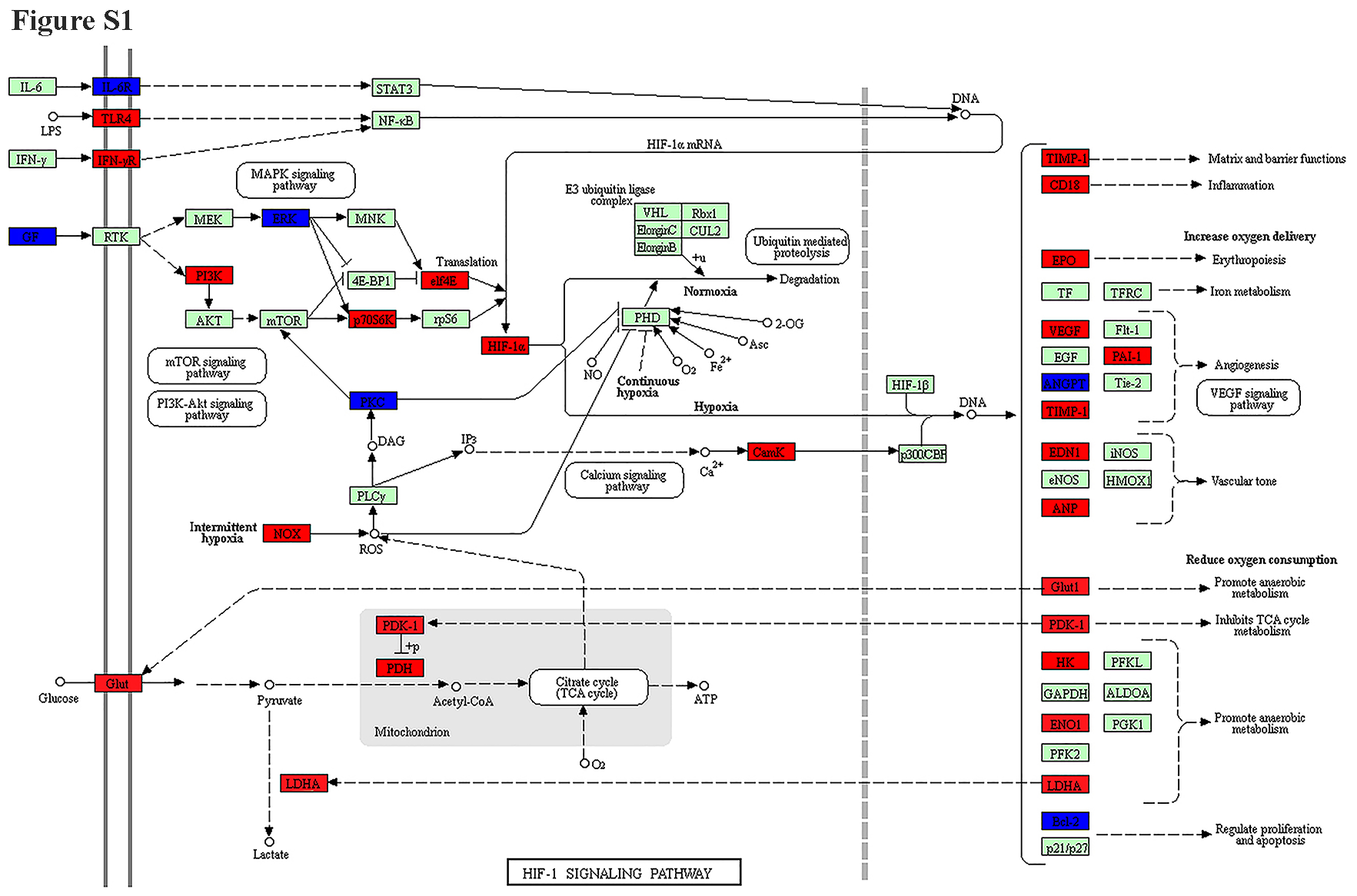

Supplement: SUPPLEMENTARY FIGURE 1 — Diagram of the HIF-1 signaling pathway. Diagram of the HIF-1 signaling pathway by Kyoto Encyclopedia of Genes and Genomes analysis. Blue means gene expressed at lower levels. Red means gene expressed at higher levels. Green means gene expressed with no significant difference. [file Image_1.TIF]
